# “They try to suppress us, but we should be louder”: a qualitative exploration of intimidation in tobacco control

**DOI:** 10.1186/s12992-023-00991-0

**Published:** 2023-11-16

**Authors:** Britta K Matthes, Raouf Alebshehy, Anna B Gilmore

**Affiliations:** Department for Health, University of Bath, Claverton Down, Bath, BA27AY UK

**Keywords:** Advocacy, Tobacco industry, Intimidation, Risks, Tobacco control

## Abstract

**Background:**

Tobacco control advocates and researchers face powerful opponents who go to great lengths to protect their interests. While threats and attacks are documented in the grey literature, research into intimidation remains scarce. Building on previous exploratory research, this study seeks to offer in-depth insights into experiences of intimidation in the global tobacco control community.

**Methods:**

Using qualitative description, we conducted a focus group and semi-structured interviews with tobacco control advocates and researchers to explore their experiences, including forms of, and responses to, intimidation, and ways forward. Data were analysed using qualitative content analysis.

**Results:**

Twenty-nine individuals from across the globe participated in the study. They reported several forms of intimidation including attacks in the media; online harassment; legal threats; non-legal threats, including death threats; Freedom of Information requests; perceived or actual surveillance; as well as burglary and theft. Responses included non-action (i.e. ignoring attacks); withdrawal (i.e. abandoning a project, area or field); defensive adaptation, for example through self-censorship; and offensive measures, including exposing attacks or filing complaints. Responses were shaped by several factors, including type and level of support from within internal and external networks; as well as an individual’s mindset, skills and experiences; and state-civil society relations. Participants suggested several measures that could help address intimidation: 1) report and monitor intimidation; 2) (better) prepare individuals through awareness raising and training (e.g. IT security, legal); 3) support those in need through legal advice, a peer-support network and involvement in response; and 4) look beyond tobacco control to learn and build connections.

**Conclusion:**

Intimidation is a significant challenge to tobacco control that needs urgent attention. This study suggests measures to address intimidation that require commitment from, and collaboration amongst, multiple actors including governments, international organisations, funders, researchers and civil society. Moreover, collective action beyond tobacco control is needed to not only manage but move beyond intimidation.

## Introduction

There is a growing recognition that commercial actors, their products and practices impact our health [1]. In a globalised, neoliberal system, in which some corporations have higher revenues than many states [2], the products from four industries – tobacco, ultra-processed food, alcohol and fossil fuels – account for at least one in every three global deaths [1]. Those exposing the corporate practices of unhealthy commodity industries (UCIs) and advocating for tighter regulation, have powerful opponents who go to great lengths to protect their interests (i.e., profit maximisation), including seeking to influence policy-making [3], manipulating science [4] and public opinion [5], and marketing their products to vulnerable groups [6, 7].

A recent taxonomy of corporate political activity highlights that UCI actors seek to weaken their adversaries in order to build a corporate-friendly policy environment [3]. To do so, they monitor the public health community, instigate fragmentation, and attack and defame its members [3]. Several such instances have been documented, mostly in the grey literature, ranging from public attacks on reputation [8, 9] to legal threats and action [10, 11], surveillance [12–14], physical threats [10, 15, 16] and attacks [17]. In such cases, public health progress can be stalled or undermined given the public health community’s vital role in pushing for strong and effective policies and countering unnecessary industry interference. Nevertheless, hardly any research has focused on intimidation in the public health community, its prevalence or its impact.

A notable exception is our recent exploratory study on experiences and perceptions of intimidation among members of the tobacco control community based in low- and middle-income countries (LMICs) [18]. Over two thirds of participants of the small-scale survey reported that they or a colleague had experienced intimidation in relation to their tobacco control work [18]. The forms of intimidation, listed in Table 1, varied from overt (i.e. public-facing) to more covert (i.e. hidden, less visible to others) ones. Table 1 also includes frequency of reporting, indicating that intimidation affects advocates and researchers across all five WHO regions covered by the survey (the sixth WHO region, Western Pacific, was not represented in the sample).

**Table 1:** Forms of intimidation, adapted from Matthes et al. (2022) [18]

Form of intimidation	% of participants who reported that they or a colleague had experienced intimidation	Number of WHO regions in which the intimidation was recorded
Public discreditation—social media	61%	Five
Public discreditation—traditional media	52%	Four
Public discreditation—other (e.g., statements on a website)	48%	Four
Legal threats or attacks	48%	Four
Non-anonymous intimidating messages	43%	Five
Anonymous intimidating messages	39%	Four
Cyberattacks	34%	Four
Physical violence/intimidation	17%	Three
Theft/burglary	13%	Three
Spying/surveillance	9%	Two
Complaint to the employer	9%	One
Disseminating false information about individual/organisation among policy makers/in hearings	9%	Two
Complaint to controlling authority	4%	One

Revealing impacts on organisations, their resources and relationships with relevant stakeholders, the study suggested that intimidation is indeed a critical challenge to tobacco control progress. Threats and attacks also affect individuals and have even driven some away from tobacco control, which costs the community in terms of capacity.

The study called for more work in the area given its limited scope and size. Partly because of the COVID-19 pandemic, the study was mostly survey-based, which did not allow for the capture of in-depth insights into individuals’ experiences. The study also only focused on LMICs despite intimidation being a global phenomenon. With this follow-up study, we aim to address these shortcomings and explore experiences of intimidation among tobacco control advocates and researchers across all six WHO regions and countries, irrespective of income level, looking at 1) what forms of intimidation they face, 2) how they respond to intimidation, 3) what informs their response and 4) what could be done to address intimidation in tobacco control, in order to strengthen the community and promote public health.

A more in-depth understanding of intimidation in tobacco control could help identify ways forward, and the extent to which these could build on existing efforts. The World Health Organization Framework Convention on Tobacco Control (WHO FCTC) [19] provides a roadmap for global tobacco control. Its Conference of Parties, which takes the decisions necessary to promote the treaty’s effective implementation, has recognised the crucial role of civil society in tobacco control, but until today there has not been a recommendation regarding intimidation.

Given the similarity of industry strategies across UCIs [3], the study also seeks to spark discussions among all those working for public health, and facing corporations that threaten the progress of public health.

## Methods

As in the previous study, our understanding of intimidation – “*actions that make you feel frightened or threatened*” – focused on the perception of individuals [18]. Given the focus on individuals’ experiences and views on ways forward, we opted for a qualitative descriptive approach [20] using a focus group and semi-structured interviews.

### Sampling and recruitment

We purposely sought to select focus group and interview participants with different roles, levels of experience and active in all parts of the world, and in countries of all income levels.

For the focus group, participants needed to be fluent in English and for the interviews, they needed to speak English, German or Spanish so that the lead researcher could conduct the interviews. To identify potential participants, we used our networks and snowball sampling.

### Data collection

Focus group and interview schedules were developed in a series of author meetings. They covered participants’ experiences of intimidation, their responses, and what they considered necessary for addressing the issue. We offered in-person focus groups and in-person or remote interviews, arranged at participants’ convenience. Given the sensitivity of the topic, focus groups might not appear the obvious choice for data collection since – unlike in one-to-one interviews – privacy and confidentiality can be compromised [21]. However, focus groups can be empowering, allowing participants to exchange experiences and think together about solutions to a problem [22].

Data collection took place in the second half of 2022. The focus group was facilitated by the lead author, another author took notes. The interviews were conducted by the lead author. Focus group and interviews were recorded and transcribed with one exception: one interviewee did not give consent to recording the interview. Here, the interviewer took detailed notes.

### Data analysis

Transcripts and notes were analysed using qualitative content analysis, a versatile approach well-suited for studying multifaceted issues [23, 24]. NVivo (Lumivero) was used to facilitate analysis. For the forms of intimidation, the starting point were those identified in the previous study, but we remained open to identifying new types [18]. Given the qualitative approach of the study, we do not report numbers but describe and summarise reported experiences. Since the literature on responses to intimidation and ways forward is limited and fragmented, an inductive approach, also called conventional content analysis [24], was used to explore the data in relation to these research questions. First, data were coded openly [23]. Next, codes were grouped and categories were developed, each named with content-characteristic words [23]. The content of each category was summarised, including illustrative examples and quotes.

### Ethics

Ethical approval was obtained from the University of Bath's Research Ethics Approval Committee for Health (REACH) [Reference: EP 22 054].

To ensure a high standard of digital security, we encouraged potential participants to contact us via an encrypted messaging app. Due to safety concerns, we also did not offer online focus groups.

All participants gave consent to take part in the study. Given the sensitivity of the topic, participants could decide whether to allow us to report basic demographic information. If they had given us permission to do so, we indicate role and WHO region they work in when using direct quotes.

## Results

### Sample

We conducted one focus group with eight participants which lasted 112 min, and 21 individual interviews lasting between 40 and 77 min with an average duration of 56 min.

Of the 29 participants, five preferred not to share demographic data. The roles of the remaining 24 participants, their regions of activity and income-level of the countries of activity are summarised in Table 2.

**Table 2:** Demographic information of focus group and interview participants

	**Descriptors and numbers of participants**	**Prefer not to say/ not applicable**
**Role** **(self-identified)**	Advocate (A): 10 Advocate and researcher (A&R): 8 Researcher (R): 5 Advocate and journalist (A&J): 1	5 (prefer not to say)
**Region of activity**^a^	Eastern Mediterranean region (EMR): 5 from 5 countries European region (EUR): 4 from 4 countries African region (AFR): 3 from 3 countries Region of the Americas (AMR): 3 from 3 countries Western-Pacific Region (WPR): 3 from 2 countries South-East Asia (SEAR): 2 from 2 countries Global level: 4	
**Income level of country of activity**^b^	Low income: 3 Lower-middle income: 10 Upper-middle income: 4 High income: 3	5 (prefer not to say) + 4 (n/a as active at global level)

^a^Source: WHO Classification; ^b^Source: World Bank [25]

### Forms of intimidation

Participants reported a wide range of forms of intimidation.

*Public-facing attacks* seeking to “give somebody a bad name” (A/AFR) and “create a negative opinion regarding one’s efforts” (A&R/EUR) were repeatedly mentioned. This included newspaper articles criticising organisations and funders, making false claims about funding sources, or misquoting an organisation’s work. In response to a research paper, a tobacco company published a letter which “not only criticised the research, but also criticised [the author] as a bad researcher” (R/EUR). An advocate shared that “almost any time we’ve released a major report, we’ll get a response, usually [claiming] unfactual things” (A/Global).

Members from a civil society organisation discovered posters portraying them “as agents of Big Tobacco and enemies of local communities” (A&R/SEAR).

Harassment on social media was seen as a growing concern, particularly around e-cigarettes and heated tobacco products, and the topic of tobacco harm reduction. In one instance, after talking on TV about e-cigarettes, a participant received “hundreds of threatening messages” online (anonymous). Attacks also involved legal threats and derogatory memes. Participants also reported attempts to impersonate them online and the creation of fake webpages.

Several participants faced *legal threats* from tobacco companies or third parties, usually in the form of letters sent to them, their employers or journal editors. An advocate explained “they sound like cease-and-desist letters but they’re not, they don’t really have that legal backing” (A/Global). No participant reported legal action against them.

Advocates and researchers also received *non-legal threats*, including anonymous calls and messages with threats of physical violence or death. In one case, it remained unclear how personal numbers had been obtained. In another instance, a tobacco company threatened to “go after [a researcher’s] job” if they continued their work (A/AMR). Elsewhere, organisations pushing for policy change, received calls from tobacco retailer associations threatening:

*if [you] don’t stop, the association people and all the vendors will come and sit in front of [your] offices and homes, they will sit there in protest and shout slogans (R/SEAR).*

Intimidating *physical action, burglary and theft* were also mentioned. In some places, the waste disposed of by organisations and individuals was searched. In other instances, the car of an investigator and the offices of organisations were broken into. In both times, computers were stolen, and no culprit was identified.

Participants also voiced suspicions of *being followed physically or online*. For example, industry staff attended tobacco control events, in person and online, and an unknown individual took photos during government-organised workshops. Industry staff, and individuals suspected to be industry-linked, also attempted to connect with participants, for example, via LinkedIn, Facebook, email or in person. A few participants had evidence that they were under *surveillance:* “colleagues […] found that in [company] reports, they say my name” (A&R/EUR). Participants also reported hacking, or the suspected hacking, of websites and email accounts.

A few researchers reported that their employer received large numbers of *Freedom of Information requests*. Through such requests – in some jurisdictions called Access to Information requests – citizens or residents can obtain documents held by public bodies, including public universities. Participants reported “vexatious requests” relating to their work. A participant concluded: “[such requests] are done in order to try and undermine our work or to slow us down” (R/EUR).

### Responses to intimidation

Participants reported undertaking several activities in response to intimidation, which were grouped into four categories. A response could include activities across multiple categories. For instance, one could expose an attack (Offensive action) while also enhancing digital security (Defensive adaptation).

#### Non-action

Several participants noted that they or colleagues did not respond to intimidation, often referring to general ‘non-engagement’ practices. After being approached repeatedly via phone and in person, which made them feel uncomfortable, a participant recalled ignoring a company’s invitation to visit their factory (anonymous). In another instance, after a threatening call and suspected surveillance, a participant reported carrying on as planned and “nothing happened” (A/AMR). Non-action generally served to avoid “adding fuel to the fire” (R/EUR) or “create[ing] a bigger deal… than necessary” (A/Global).

#### Withdrawal

Projects can be abandoned due to safety concerns. Advocates were advised “not to try to do anything if you don’t think it’s safe” (A&R/WPR). In one instance, after researchers heard they were under surveillance, they decided to not go ahead “because we felt we couldn’t keep our people safe […], sometimes you have to walk away” (A&R/Global).

Some individuals and organisations would also move towards less risky areas such as awareness raising, or to other geographical regions. An advocate explained “for our next round of funding, we are avoiding a certain [geographical] area, [we] are fleeing” (A/WPR). Intimidation also drives individuals away from tobacco control:

*they go to other areas where they are safe. [When] advocating for malaria or HIV, you wouldn’t have anybody threatening you, following you or trying to check your emails and stuff (A/WPR).*

A participant mentioned a researcher who was repeatedly threatened and physically intimidated, “I haven't heard about any other work that [they have] done. And [they were] a very important researcher” (A/AMR).

#### Defensive adaptation

Another response was adaptation. This involved self-censorship which a participant described as “actually quite normal in tobacco control” (A&R/SEAR). Members of the tobacco control community would select their words very carefully. Reflecting on their work pushing for a bill, an advocate stated:

*[w]hen you are labelled the way they label you, you begin to withdraw some of the things that you say in public. You don't want to be somebody that is hated […] because when you make comments, the next day somebody is in the radio saying, XYZ said, which you have not (A/AFR).*

Another participant observed that some organisations “adopt a narrative which appears more legitimate for governments” (R/SEAR). More generally, changing a narrative could lead to “watering something down” (R/EUR) or weakening the message.

Intimidation also led to the adaptation of projects. For example, after researchers saw “very threatening posters”, they cut their visit to tobacco factories short: “like [a] guerrilla study [ …], before company agents received any news [about the visit], we talked [to] workers and got out” (A&R/SEAR).

Participants also reported taking other precautionary measures, including enhancing IT security, such as changing passwords regularly, using two-factor authentication, and using encrypted emails; only answering calls from known numbers; blocking social media users; getting a legal expenses insurance; working with multiple affiliations; and requesting legal review prior to publication. In these cases, participants perceived instances of intimidation as opportunities to improve practice.

#### Offensive action

Participants also reported pro-active responses to intimidation. These involved exposing threats and attacks in the media or on social media. Such actions were seen as improving safety: for example, making a death threat public would make a physical attack less likely. However, exposing intimidation in the media, “can give [aggressors] the platform to raise more voice” (A&R/WPR).

Some participants reported opting for a more targeted approach focusing on key stakeholders. For example, when newspaper articles attacked tobacco control organisations, advocates approached public officials, seeking to correct industry claims (A&R/WPR). When faced with false allegations, a researcher tried to “meet authorities to clarify the [tobacco company’s] intent. To say to [them], we are being misunderstood” (R/SEAR). Similarly, attacks were reported to employers and funders to make them understand that their purpose was to silence advocates and researchers.

Where those behind the attacks were known, complaints and legal action could be used. For example, following threats from a third party, advocates complained to the local police about the organisation (R/SEAR). Elsewhere, advocates took legal action: “[we] applied to the court to defend our rights, then our lawyer raised a complaint against those journalists, who created and distributed misleading information” (A&R/EUR).

Finally, evidence of attacks was also used for advocacy. A participant who had received derogatory and threatening messages, used these “to tell people how the industry is threatening people who are not talking in the same way they are talking” (anonymous).

### Factors informing responses

Several factors influenced responses to intimidation. which were grouped into five interlinked and partially overlapping categories.

Firstly, whether evidence on the incident and those behind it was available. Some participants reported that to speak up or complain to the police, one would need evidence; without it, concerns could only be shared with colleagues.

Secondly, internal networks within organisations were crucial in shaping responses. The backing of colleagues was key. A participant shared that “having the support from [senior colleagues] felt really important, I felt like they wouldn't let me hang dry” (A/AMR), enabling them to continue their work. Another participant reported not being taken seriously by colleagues, which made them consider leaving the field (anonymous).

It was similar for employers: a few participants felt supported by them. In one case, the head of the organisation recognised the importance of their tobacco control work, leading to “inbuilt protection” (A&R/EMR), helping industry-critical activities to go forward. Other participants expressed concerns about employers having limited resources and “not wanting to be involved in complications” (A&R/WPR). In one instance, when discussing a project on industry interference, the employer appeared “far more worried about reputational and financial risks than the public health benefits of the information” (A&R/Global).

Thirdly, external networks, including funders, lawyers, media, and international organisations, also shaped responses. Some advocates said they were able to discuss attacks with their funder and received support and advice. In one instance, after an investigator was threatened and their computer was stolen, the funder “took the pressure off”, telling them they were not expected to publish the work (A/Global). At times, funders also helped with obtaining money for legal advice. Other participants reported lacking such support: “Our funds were earmarked, you couldn’t just say: We now have a legal problem. You better don’t mention it.“ (A&R/EUR). Another advocate worried that intimidation “c[ould] affect the flow of donor support” (A/AFR).

Connections with lawyers with an in-depth understanding of the tobacco industry was also important. They could, for example, assess whether a legal letter just sought to scare the recipient. But not all advocates and researchers had such contacts. Similarly, some found collaborating with journalists to expose intimidation very helpful, but others lacked such connections. International organisations were also contacted for support. An advocate recalled that obtaining a support letter was “very, very difficult [and took] a long time” (A/EMR) and another was asked: “why don’t you do something else in tobacco control?” (R/SEAR).

Fourthly, individual factors, including mindset, skills and previous experiences also informed responses. Many participants framed intimidation as an indicator of impact. In the words of one participant: “[attacks] give me the assurance that I'm on the right track” (anonymous). When faced with intimidation, participants also reported reminding themselves of their aims and values, one recalled “remember[ing] what [they] believe in” (A&R/Global). Responses were shaped by skills such as the ability to think ahead and conduct thorough risk assessments. A researcher found that “[i]t’s like chess, you have to always think five steps further” (R/EUR). Another researcher shared they tried to read everything from an industry perspective before publication, seeking to anticipate potential responses (R/EUR). Yet, other participants were less confident. A researcher stated “I don’t have skills to protect myself and my family. So, I keep [information about industry conduct] to myself.” (R/WPR).

Previous experiences also informed responses. The first incident was often perceived most challenging: “I specifically remember where I was when I [received the first legal threat]” (R/EUR). With every incident, they would learn and generally cope better.

Lastly, state-civil society relations also mattered. For some, the state could offer support, for example, with regulation “protect[ing] us against the misuse of personal information” (anonymous). The ministry of health was also seen as source of (potential) support (R/EMR), yet elsewhere, it was perceived “the weakest ministry with no budget” (A&R/WPR).

Some participants expressed concerns about repression against civil society more generally. An advocate explained that “[civil society’s] relationship with the government is not good […], there are just a few spaces in which we can interact [and] the space is shrinking” (A/AFR). It was also observed that governments were increasingly “buying a lot of narrative from the [tobacco] industry and coming down on civil society” (R/SEAR). Close government-industry links were a major concern. In such scenarios, “there’s just not that same rule of law and level of protection for individuals” (A/Global).

### Ways forward

Participants identified several measures to address intimidation, with the responsibility lying with all tobacco control actors.

#### Report and monitor intimidation

It was perceived important to document intimidation: “We actually never thought of recording these cases of intimidation very seriously… But we should” (R/EUR). An advocate explained: “incidences [are] happening in each country […] but in the end, you look at them, they are repeating” (A/EMR). Through monitoring, patterns could be recognised and effective responses identified (A&R/Global).

#### Better preparation

In order to better prepare individuals and organisations, raising awareness was considered key. As a participant explained: “we hear a lot about industry interference, what kind of risks it puts on people is not talked about” (A&R/EUR). In this, it would be helpful for individuals to also realise that “not all of it leads to disastrous consequences, part of it is to intimidate you” (R/SEAR).

Specific training could also help. This should cover digital security: “lots of people expose their information just because they don't know [the risks]” (A/AMR); and libel, covering “what is defensible and what’s not, at a basic level” (R/EUR). Participants also expressed the need for training on documenting and handling intimidation. Any capacity building efforts could involve “practical examples and hands-on actionable advice” (A&R/EUR). While training could be part of project funding, participants also thought they should be accessible to all, for example, as online modules (A/Global).

#### Support those in need

More support or “a safety net” (A/EMR) was seen as needed for those in challenging situations. Access to legal advice and support from lawyers familiar with industry tactics would be crucial to check legal threats, draft response statements and “be a fallback in case [one] actually do[es] get sued” (R/EUR). A “network of high-level lawyers” (A/AMR) or “a central legal team” (R/SEAR) offering pro-bono support could be created and money for legal support could be set aside in each grant.

Furthermore, providing a safe space where individuals can voice their fears and concerns, and discuss next steps, would be important. Reflecting on their own experiences, a participant found that “just knowing you’re not alone is a big part” (A&R/WPR). Speaking to someone with lived experiences would be particularly valuable as they could empathise and offer reassurance. A participant explained:

*you need colleagues who’ve been working on this industry, or similar industries, for a long time, who actually understand what is a real risk, what is a perceived risk, and also how to deal with it (A&R/Global).*

In the case of public attacks, support could also involve others speaking up on one’s behalf, writing a collective response or publicising the incident internationally. An advocate mentioned that “having a global response like ‘We say that’s a bad thing’ could be really impactful” (A/Global), pressuring governments to act. Furthermore, someone experiencing an online firestorm, could be supported with positive messages (a ‘lovestorm’) (A&R/EUR). To be effective, all such interventions would need to be timely.

#### Look beyond tobacco control

Participants also felt that we could learn from other spaces in which intimidation happens. This could involve considering measures taken to enhance safety (e.g. secret meetings, buddy-systems) (A&R/EUR) and exploring existing tools for support, including The UN Declaration on Human Rights Defenders—“Health is a human right, so we are of course human rights defenders!” (A/AMR). One could also seek to connect (more) with those working in other sectors with powerful corporations, learning from each other’s experiences and building alliances (A&R/Global).

## Discussion

This is the first study to offer insights into intimidation in the global tobacco control community, exploring forms of intimidation, responses and ways forward. In line with our recent LMIC-focused research [18], this study suggests that advocates and researchers experience several forms of intimidation, supporting our argument that intimidation is a key tobacco control challenge. Public-facing attacks were reported repeatedly, and online harassment was perceived as particularly concerning. Legal threats to advocates, researchers, employers or editors appeared common but in no case was legal action reported as being taken against them. There were also threats of job loss, violence and even death. Advocates and researchers also felt watched, either physically or online. Physical intimidation, break-ins and theft appeared – as in the previous study [18] – rare. Complaints to employers and controlling authorities were mentioned infrequently before [18] and not at all here, which could mean it is indeed a rare occurrence, potentially highly context-dependent. Freedom of Information requests were the only type of intimidation not mentioned before, potentially because of the small sample size of the previous study.

This research further illustrates the responses to intimidation. These ranged from not acting to abandoning a project or area of work, and from taking defensive measures such as self-censorship to offensive action, for instance, calling out industry conduct and filing complaints. Responses were informed by several interlinked factors. Colleagues, employers and funders could provide reassurance and at times ad-hoc support. Also, legal and media connections and international organisations could help. But other individuals lacked such back-up, were not taken seriously, or could not discuss their concerns with employers or funders. This could lead to them moving away from tobacco control which would mean a loss for the community. In addition, state-civil society relations mattered: for example, where individuals do not feel protected by state institutions and officials, their responses could be more timid.

Finally, the study identified ways of addressing intimidation. First, instances of intimidation would need to be recorded and monitored. Second, individuals would need to be better prepared through awareness raising and specific training. Third, better support was considered necessary for those in challenging situations, including access to legal advice and peer support. Fourth, there are opportunities to learn from other fields, explore potential support mechanisms (e.g., UN Declaration on Human Rights Defenders), and build connections.

This study has a number of limitations. First, participants were recruited through our networks and self-selected, meaning that the sample is not representative of the tobacco control community. Individuals who experienced intimidation were potentially more likely to participate. The sample is also not evenly spread across regions which could link to language barriers. Second, we draw on participants’ insights, meaning that recall and social desirability biases cannot be ruled out. To limit the latter, we used established strategies, including providing assurances, prefacing key questions with context and using probes to identify examples and obtain more information [26]. Third, focus group participants might not have felt they could talk freely [27]. To address this, we invited participants to contact us afterwards if they wanted to share further insights.

Despite these limitations, key findings resonate with the wider literature. A recent study found that online self-censorship has increased among political activists in recent years [28]. In our study, self-censorship was a common response to intimidation. This is alarming as it prevents freedom of expression, and access to, and flow of, information [29]. A participant described self-censorship as “quite normal”, which echoes findings on journalists working in a crisis-ridden country who internalise self-censorship [30]. Elsewhere, it was emphasised that while limiting freedom, self-censorship can also help create spaces in which journalists are able to work [31]. Recent contributions highlight the importance of the political context [32–36] which could also be explored further, for example, looking at country- or regional level.

A recent commentary on journalists’ safety, mentioning forms of intimidation similar to those reported here, raises important concerns that resonate with our findings: that organisations usually lack resources to provide training and legal support, and ensure digital safety [37]. Another study identified a “pressing need” for journalist education to avoid foreseeable risks [38]. Furthermore, precarity and freelancing can compromise safety [37], which links to our findings that those without a funder and/or resources lack important sources of potential support. Another concern, also raised in our study and in relation to human rights defenders [39], was that many states lack effective policies to prevent attacks and bring culprits to justice [37].

The proposed measures to address intimidation also link to existing research: a study on online harassment among female activists and journalists recommends documenting attacks and sharing the evidence in support networks [40]. This approach encompasses measures identified here (reporting and monitoring, raising awareness) but also highlights how these could help recovery [40], which could potentially prevent individuals from abandoning their work – which was a concern raised in this study.

Considering the idea of looking beyond tobacco control, several initiatives are of interest. A wide range of materials and toolkits about safety and security exist for activists [41, 42] and journalists [43, 44] facing powerful opponents. For example, Front Line Defenders [45] offer, among others, a ‘Workbook on Security’ for individuals and organisations to develop a security plan and the International Press Institute [46] provides a detailed website on online harassment, aiming at supporting newsrooms in building a protocol to address this.

Reporting and monitoring systems – suggested here as a way forward – exist for human rights defenders, including activists and journalists generally (e.g. Business & Human Rights Resource Centre [47], Front Line Defenders [48]), and those in specific areas (e.g. Global Witness [49]). Furthermore, the Committee to Protect Journalists [50] documents cases of media workers killed around the world, and the International Federation of Journalists [51] regularly reports on abuse and attacks.

Peer-support initiatives have also been established, for example, for climate activists [52] and journalists working for Reuters and the BBC [53]. Furthermore, emergency funds, grants and fellowship schemes exist for human right defenders, including some aimed at journalists and academics [54].

This study calls for action. The Parties to the WHO FCTC could recognise intimidation as a challenge for tobacco control and decide on actions to protect all those working towards achieving the treaty’s aims. International organisations could adopt a scheme allowing those in need to access legal support, especially those with limited/no resources and support. The community should consider developing a system for reporting and monitoring intimidation, which could build on or link to existing initiatives, and establish a peer-support network. Funders could ensure that risks are always considered, for example, as part of applications and early discussion, and training should be funded where needed. More generally, resources and training should be developed – potentially learning from initiatives in other areas – and/or made widely accessible, so that not only a few benefit [55].

While all these measures could help individuals and organisations to better cope with intimidation, more is to be done. Intimidation is underpinned by wider systems dynamics, including large power and resource discrepancies [1]. We should not accept intimidation as a necessary evil we need to manage, but collectively work towards broader transformative change [56], holding actors to account and creating a world in which advocates, researchers and others can advance public health without fear and intimidation.

## Conclusion

This is the first study on experiences of intimidation in the global tobacco control community, indicating that intimidation in its multiple forms is a critical challenge for tobacco control. It illustrates that advocates and researchers use several strategies to respond to intimidation, ranging from non-action to offensive action, and from withdrawal to defensive adaptation. It also suggests a range of measures to address intimidation requiring commitment from all tobacco control actors. Moreover, collective action beyond tobacco control for systemic change is needed to not only manage but move beyond intimidation.

## Data Availability

N/a.

## Abbreviations

A: Advocate
AFR: African Region
AMR: Americas Region
A&R: Advocate and Researcher
A&J: Advocate and Journalist
EMR: Eastern Mediterranean Region
EUR: European Region
LMICs: Low- and middle-income countries
R: Researcher
SEAR: South-East Asian Region
WHO FCTC: World Health Organization Framework Convention on Tobacco Control
WPR: Western Pacific Region
